# Caspase-mediated cleavage of the exosome subunit PM/Scl-75 during apoptosis

**DOI:** 10.1186/ar2119

**Published:** 2007-02-05

**Authors:** Geurt Schilders, Reinout Raijmakers, Kelen CR Malmegrim, Lieselotte Vande Walle, Xavier Saelens, Wilma Vree Egberts, Walther J van Venrooij, Peter Vandenabeele, Ger JM Pruijn

**Affiliations:** 1 Department of Biomolecular Chemistry, Nijmegen Center for Molecular Life Sciences, Institute for Molecules and Materials, Radboud University Nijmegen, Geert Grooteplein 26–28, Nijmegen, 6525 GA, The Netherlands; 2 Center for Cell-Based Therapy, Faculty of Medicine of Ribeirão Preto, University of São Paulo, Ribeirão Preto, São Paulo, 14049-900, Brazil; 3 Department of Molecular Biomedical Research, Flanders Interuniversity Institute for Biotechnology, Ghent University, Technologiepark 927, Ghent, B-9052, Belgium

## Abstract

Recent studies have implicated the dying cell as a potential reservoir of modified autoantigens that might initiate and drive systemic autoimmunity in susceptible hosts. A number of subunits of the exosome, a complex of 3'→5' exoribonucleases that functions in a variety of cellular processes, are recognized by the so-called anti-PM/Scl autoantibodies, found predominantly in patients suffering from an overlap syndrome of myositis and scleroderma. Here we show that one of these subunits, PM/Scl-75, is cleaved during apoptosis. PM/Scl-75 cleavage is inhibited by several different caspase inhibitors. The analysis of PM/Scl-75 cleavage by recombinant caspase proteins shows that PM/Scl-75 is efficiently cleaved by caspase-1, to a smaller extent by caspase-8, and relatively inefficiently by caspase-3 and caspase-7. Cleavage of the PM/Scl-75 protein occurs in the C-terminal part of the protein at Asp369 (IILD^369^↓G), and at least a fraction of the resulting N-terminal fragments of PM/Scl-75 remains associated with the exosome. Finally, the implications of PM/Scl-75 cleavage for exosome function and the generation of anti-PM/Scl-75 autoantibodies are discussed.

## Introduction

Systemic autoimmune diseases are characterized by the presence of autoantibodies reactive to a wide variety of autoantigens. Why these autoantibodies, which escape the normal mechanisms ensuring self tolerance, are made is still not fully understood. However, the occurrence of modified self-antigens during (either apoptotic or necrotic) cell death in combination with a defective clearance of dead cells has been proposed to have a role in the development of autoimmunity (reviewed in [1,2]). In apoptotic cells many autoantigenic proteins or complexes can be modified by processes such as (de)phosphorylation, citrullination, nucleolytic cleavage or proteolytic cleavage by caspases (reviewed in [3]). The modification and redistribution of these proteins might generate antigenic determinants to which no tolerance exists, thereby eliciting a primary immune response. Via epitope spreading, the initial response, directed to the neo-epitope resulting from the modification, could evolve to a secondary response in which antibodies arise that are reactive with other, unmodified parts of the protein or with proteins that are associated with the modified antigen [1,4].

Patients suffering from myositis and scleroderma (Scl), which is called the polymyositis/scleroderma overlap syndrome (PM/Scl), produce antibodies against a variety of autoantigens. Some of these are also found in patients suffering from myositis or scleroderma alone. Autoantibodies recognizing the so-called PM/Scl autoantigen are found in 24 to 31% of all patients with PM/Scl [5-8], and in only 2 to 6% of patients suffering from myositis or scleroderma alone [7,9]. Of all patients positive for anti-PM/Scl antibodies, between 43% and 88% are diagnosed with a myositis/scleroderma overlap syndrome [7,10]. The PM/Scl autoantigen is the human homologue of the yeast exosome, which consists of at least nine core proteins, all displaying exoribonuclease characteristics. The exosome has been shown to be involved in the degradation and processing of many different RNA species [11,12]. Although the nuclear exosome component PM/Scl-100 and the two core exosome components PM/Scl-75 and hRrp4p carry the main autoantigenic epitopes, autoantibodies directed against PM-Scl-75 seem to be the most prevalent in patients with the polymyositis/scleroderma overlap syndrome [8]. The cDNA-derived amino acid sequence for PM/Scl-75 was published in 1991 and is now referred to as PM/Scl-75a-α. A splicing variant of PM/Scl-75a containing an additional exon in the C-terminal region of the protein is known as PM/Scl-75a-β [13]. More recently, we found that the PM/Scl-75a cDNA sequence is probably incomplete, and identified a PM/Scl-75 cDNA (referred to as PM/Scl-75c) encoding an additional N-terminal part that is required for association with the exosome complex [14].

Until now, none of the subunits of the exosome complex had been shown to be modified during apoptosis, prompting us to investigate the molecular characteristics of exosome subunits in apoptotic cells. Here we demonstrate that the PM/Scl-75 protein is cleaved in a caspase-dependent manner during apoptosis and that this cleavage occurs in the C-terminal domain of the protein at residue Asp369.

## Materials and methods

### Cell lines

Jurkat cells (human T-cell leukemia, ATCC CRL-2570), Peer cells (human T-cell leukemia) and CCRF-CEM cells (human T-cell lymphoblastic leukemia, ATCC CCL-119) were grown in RPMI-1640 medium (Gibco-BRL, Gaithersburg, USA) supplemented with 10% heat-inactivated fetal calf serum, 1 mM sodium pyruvate, penicillin (100 units/ml), and streptomycin (100 μg/ml). Jurkat cells stably transfected with Bcl-2 (Jurkat/Bcl-2) or with the empty transfection vector (Jurkat/Neo) were cultured in the same medium with the addition of 200 μg/ml G418 (Gibco-BRL).

### Induction of cell death

To induce apoptosis, Jurkat cells were treated with the agonistic anti-Fas monoclonal antibody 7C11 as described previously [15,16]. Peer and CCRF-CEM cells were treated with 0.5 μg/ml actinomycin D, 10 μg/ml anisomycin, 100 μg/ml cycloheximide or 400 nM staurosporin. CCRF-CEM cells were also treated with the anti-Fas antibody. The efficiency of apoptosis induction was assessed by flow cytometry with the use of staining with fluorescein isothiocyanate-coupled annexin V and propidium iodide (PI) as described previously [15]. After 8 hours generally more than 90% of the cells were apoptotic. After harvesting of the dying cells, cells were washed twice with phosphate-buffered saline and used immediately or stored at -70°C. For experiments with the cell-permeable tetrapeptide caspase inhibitors (Calbiochem, Darmstadt, Germany), Jurkat cells were cultured for 1 hour in the presence of 2 or 20 μM Ac-YVAD-CMK, Z-DEVD-FMK, Z-IETD-FMK or Z-LEHD-FMK (irreversible inhibitors of caspase-1, caspase-3/-7, caspase-8 and caspase-9, respectively) as described previously [16]. The specificity of these inhibitors is based on *in vitro *assays with purified caspases. Their specificity in a cellular context is difficult to define. Subsequently, apoptosis was induced by the addition of anti-Fas monoclonal antibody followed by harvesting the cells after 8 hours of incubation.

### Western blot analysis

Cells were lysed on ice for 30 minutes in Nonidet P40 lysis buffer (25 mM Tris-HCl, pH 7.5, 1% Nonidet P40, 100 mM KCl, 10 mM MgCl_2_, 1 mM dithiothreitol), containing a protease inhibitor cocktail (Complete; Roche, Mannheim, Germany). Cell lysates were centrifuged for 15 minutes at 4°C (12,000 *g*) and the supernatants were used immediately or stored at -70°C. Protein extracts of 10^6 ^cells were analyzed by 10% SDS-PAGE and Western blotting with systemic lupus erythematosus patient serum Ven96 (a patient serum reactive with many exosome subunits), an anti-PM/Scl-100 rabbit serum [17] or an anti-PM/Scl-75 rabbit or mouse serum [18], followed by detection by means of horseradish peroxidase-conjugated secondary antibodies and visualization by chemiluminescence.

### Immunoprecipitation

Protein A–agarose beads (20 μl of 50% slurry) were coated with 20 μl of Ven96 patient serum, anti-hRrp46p rabbit serum [17] or anti-PM/Scl-75 [18] rabbit serum. Incubations were performed overnight at 4°C in IPP500 buffer (500 mM NaCl, 10 mM Tris-HCl, pH 8.0, 0.1% Nonidet P40) by end-over-end rotation. After the beads had been washed three times with IPP150 (composition similar to IPP500, but containing 150 mM NaCl), the beads were incubated with 20 μl (2 × 10^6 ^cell equivalents) of Jurkat extract (control or apoptotic) in IPP150 by end-over-end rotation for 2 hours at 4°C. After three wash steps with IPP150, the beads were resuspended in protein sample buffer and immunoprecipitated proteins were analysed by SDS-PAGE and Western blotting.

### Plasmids

The cDNAs of PM/Scl-75a-α (GenBank accession number M58460) and PM/Scl-75c-α (accession number AJ505989) [14] were cloned into the *Eco*RI and *Xba*I sites of the pCI-neo vector (Promega, Madison, USA), containing an in-frame vesicular stomatitis virus G epitope (VSV-G) tag at either the 5' end or the 3' end of the cDNAs. For identification of the caspase cleavage site, mutant cDNAs of PM/Scl-75 were generated, encoding substitution mutants in which one of the aspartic acid residues at positions 272, 304, 307, 349, 352, 357, 358, 363, 369, 374 and 381 were replaced by alanine. All mutants were generated by a megaprimed PCR-based approach with a PM/Scl-75 cDNA as a template and specific primers overlapping the regions that were mutated. The resulting PCR products were purified, and then cloned into the pCR4-TOPO (Invitrogen, Carlsbad, USA) or pCI-neo vector. The integrity of the mutant constructs was confirmed by DNA sequencing. The resulting cDNAs were used as templates for *in vitro *transcription/translation of the respective proteins.

### *In vitro *cleavage assay

Proteins were generated by *in vitro *transcription and translation with the TnT T7-coupled rabbit reticulocyte lysate system (Promega) as described by the manufacturer. For detection of the translation products, [^35^S]methionine was added to the translation reactions. Proteins translated *in vitro *were incubated with the purified murine recombinant caspases at 200 nM in a total volume of 25 μl of CFS buffer (220 mM mannitol, 68 mM sucrose, 2 mM NaCl, 2.5 mM KH_2_PO_4_, 10 mM HEPES, pH 7.4, 1 mM aprotinin, 1 mM leupeptin, 1 mM phenylmethylsulfonylfluoride, supplemented with 10 mM dithiothreitol) for 1.5 hours at 37°C. The resulting cleavage products were analyzed by 10% SDS-PAGE and Western blotting followed by autoradiography.

## Results

### Cleavage of PM/Scl-75 during apoptosis

**Figure 1:**
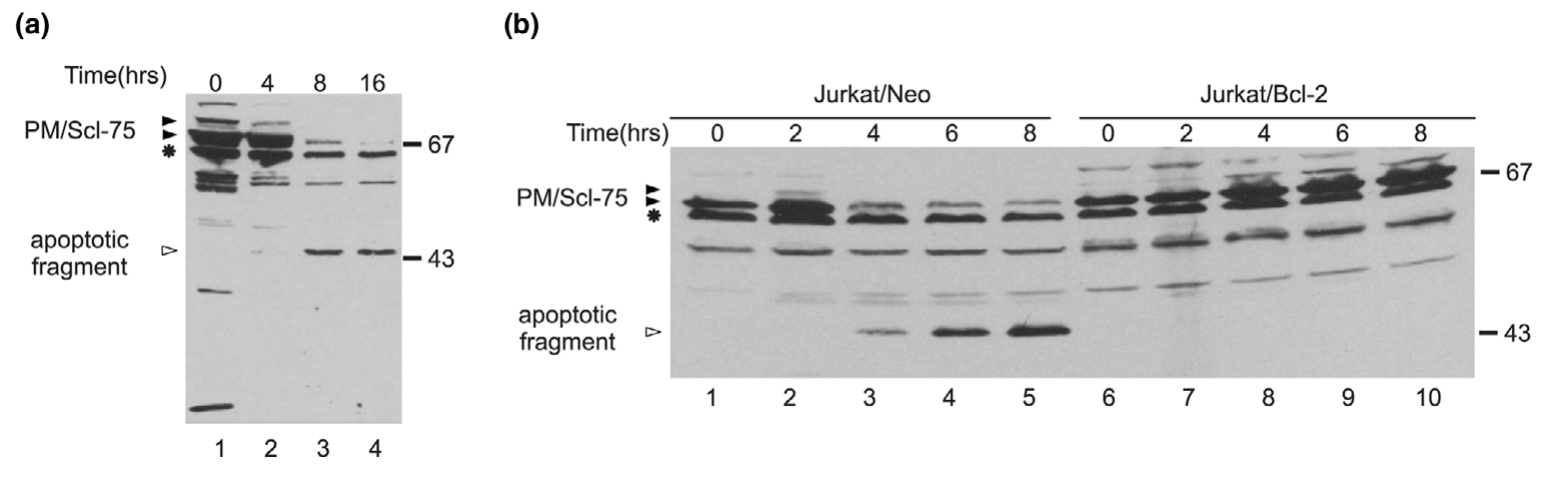
Cleavage of the PM/Scl-75 protein during anti-Fas-induced apoptosis. **(a) **Jurkat cells were treated with the anti-Fas monoclonal antibody 7C11 for the indicated durations. Protein extracts were analyzed by 10% SDS-PAGE and Western blotting with a polyclonal anti-PM/Scl-75 rabbit serum. **(b) **Jurkat/Neo and Jurkat/Bcl-2 cells were treated with the anti-Fas monoclonal antibody 7C11 for the indicated durations. Protein extracts were analyzed by 10% SDS-PAGE and Western blotting with the anti-PM/Scl-75 rabbit serum. In each panel the positions of molecular mass markers are indicated in kDa at the right and the positions of the relevant polypeptides are shown at the left. Filled arrowheads indicate full-length proteins, open arrowheads indicate cleavage products and the asterisk indicates a cross-reactivity of the anti-PM/Scl-75 serum with an unknown protein.

To study the potential modification of human exosome components during apoptosis, Jurkat cells were treated with the agonistic anti-Fas antibody 7C11 and cell extracts were analyzed by Western blotting with a patient's serum (Ven96) known to be reactive with the major autoantigenic exosome components PM/Scl-100, PM/Scl-75 and hRrp4p. The results of this analysis demonstrated that the intensity of the PM/Scl-75 band was strongly reduced after the induction of apoptosis, whereas the signals of PM/Scl-100 and hRrp4p were only slightly diminished (data not shown). To analyze this phenomenon in more detail, a similar blot was analyzed with a rabbit serum raised against PM/Scl-75 (Figure 1a). The result confirmed that most full-length PM/Scl-75 disappeared during apoptosis, concomitant with the appearance of a faster-migrating polypeptide at approximately 45 kDa (Figure 1a, lane 3). To investigate whether the production of this putative PM/Scl-75 cleavage fragment was indeed dependent on the induction of apoptosis, the cleavage of PM/Scl-75 was analyzed in anti-Fas-stimulated normal Jurkat cells (Jurkat/Neo) and Jurkat cells overexpressing Bcl-2 (Jurkat/Bcl-2). In the Jurkat/Bcl-2 cells the induction of apoptosis has been demonstrated to be delayed considerably by preventing cytochrome *c *release and caspase activation [15]. Indeed, the PM/Scl-75 cleavage product was produced in the Jurkat/Neo cells but could not be detected in the Jurkat/Bcl-2 cells, not even after 8 hours of anti-Fas treatment (Figure 1b).

**Figure 2:**
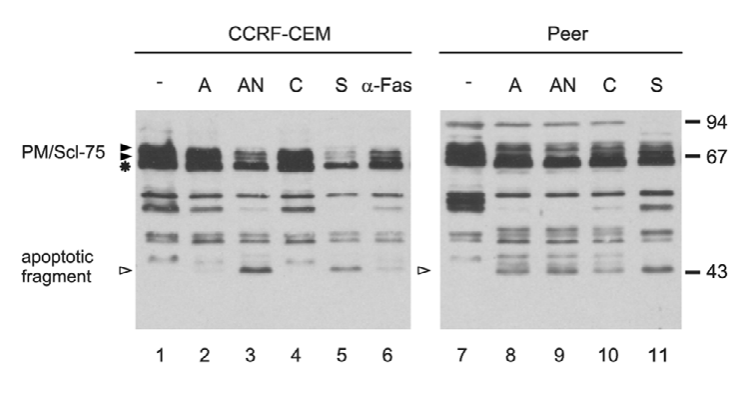
Cleavage of the PM/Scl-75 protein in various cell types treated with different apoptosis inducers. CCRF-CEM cells were treated with actinomycin D (A), anisomycin (AN), cycloheximide (C), staurosporin (S) or anti-Fas mAb 7C11 (α-Fas); Peer cells were treated with actinomycin D (A), anisomycin (AN), cycloheximide (C) or staurosporin (S). Cell extracts were analyzed by 10% SDS-PAGE and Western blotting with the anti-PM/Scl-75 rabbit serum. Extracts from mock-treated cells were loaded in lanes 1 and 7. The positions of molecular mass markers are indicated in kDa at the right and the positions of the relevant polypeptides are shown at the left. Filled arrowheads indicate full-length proteins and open arrowheads indicate cleavage products; the asterisk indicates a cross-reactivity with an unidentified protein.

To investigate whether cleavage of PM/Scl-75 also occurred in other cells and on exposure to other apoptotic stimuli, we analyzed lysates of other cell lines as well as lysates from lymphocytes isolated from peripheral blood in which apoptosis had been induced in various ways. CCRF-CEM cells were exposed to actinomycin D, anisomycin, cycloheximide, staurosporin or anti-Fas antibodies and Peer cells were cultured in the presence of actinomycin D, anisomycin, cycloheximide or staurosporin, and after 8 hours extracts of these cells were analyzed by Western blotting (Figure 2). Except for the CCRF-CEM cells treated with actinomycin D (lane 2) and with cycloheximide (lane 4), a decrease in the full-length PM/Scl-75 signals and the appearance of the cleavage product of PM/Scl-75 was detected in all lysates, indicating that PM/Scl-75 cleavage is a general phenomenon in apoptotic cells. As can be seen in Figures 1 and 2, the anti-PM/Scl-75 rabbit serum also showed reactivity with a number of as yet unidentified proteins. Apoptotic modification of PM/Scl-75 was also observed in the lysates from the lymphocytes on treatment with anti-Fas antibodies or ionomycin for 6 hours (data not shown).

### Association of the PM/Scl-75 cleavage fragment with the exosome complex

**Figure 3:**
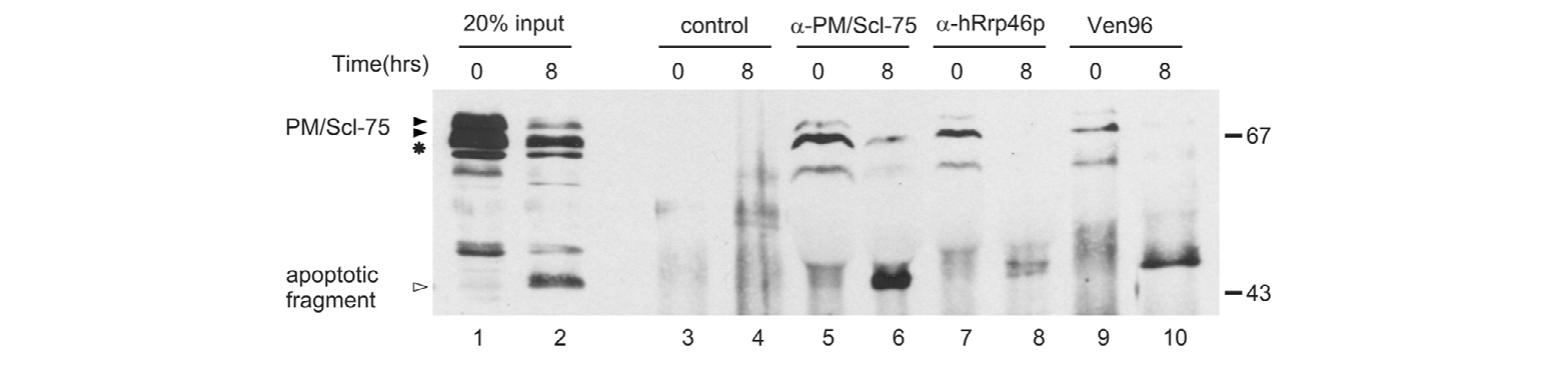
The apoptotic cleavage product of PM/Scl-75 remains associated with the exosome. Immunoprecipitations from control (0 hours) and apoptotic (8 hours incubated with anti-Fas antibody) Jurkat cell extracts were performed with normal rabbit serum (lanes 3 and 4), anti-PM/Scl-75 (lanes 5 and 6) and anti-hRrp46p (lanes 7 and 8) rabbit sera, and patient serum Ven96 (lanes 9 and 10). In lanes 1 and 2 input samples, 20% of the total control and apoptotic cell extracts, respectively, were loaded. Immunoprecipitates were analyzed by 10% SDS-PAGE and Western blotting with a polyclonal anti-PM/Scl-75 mouse serum. The positions of the PM/Scl-75 protein (filled arrowhead), its cleavage product (open arrowhead) and a cross-reactivity of the anti-PM/scl-75 serum (asterisk) are indicated at the left. The positions of molecular mass markers are indicated in kDa at the right.

The most obvious explanation of the results described above is that PM/Scl-75 is cleaved by a protease that is activated in apoptotic cells, resulting in two proteolytic fragments. Because the rabbit antiserum is reactive only with the 45 kDa fragment, our data do not exclude the possibility that the other fragment is further degraded. PM/Scl-75 is one of the core components of the exosome complex, and most PM/Scl-75 molecules in a cell are believed to be associated with this complex. To analyze whether the apoptotic cleavage fragment of PM/Scl-75 can associate with the exosome complex, immunoprecipitations were performed with extracts from control and apoptotic Jurkat cells. The results in Figure 3 show that not only anti-PM/Scl-75 rabbit serum and patient serum Ven96, but also rabbit antibodies against hRrp46p, another exosome core subunit, (co-)precipitated both the wild-type and the cleaved PM/Scl-75 protein. Note that in this experiment PM/Scl-75 was detected by a polyclonal mouse serum raised against the recombinant protein. This result indicates that at least a subset of cleaved PM/Scl-75 molecules either binds to the exosome or remains associated with the core of the exosome.

### PM/Scl-75 cleavage is caspase mediated

**Figure 4:**
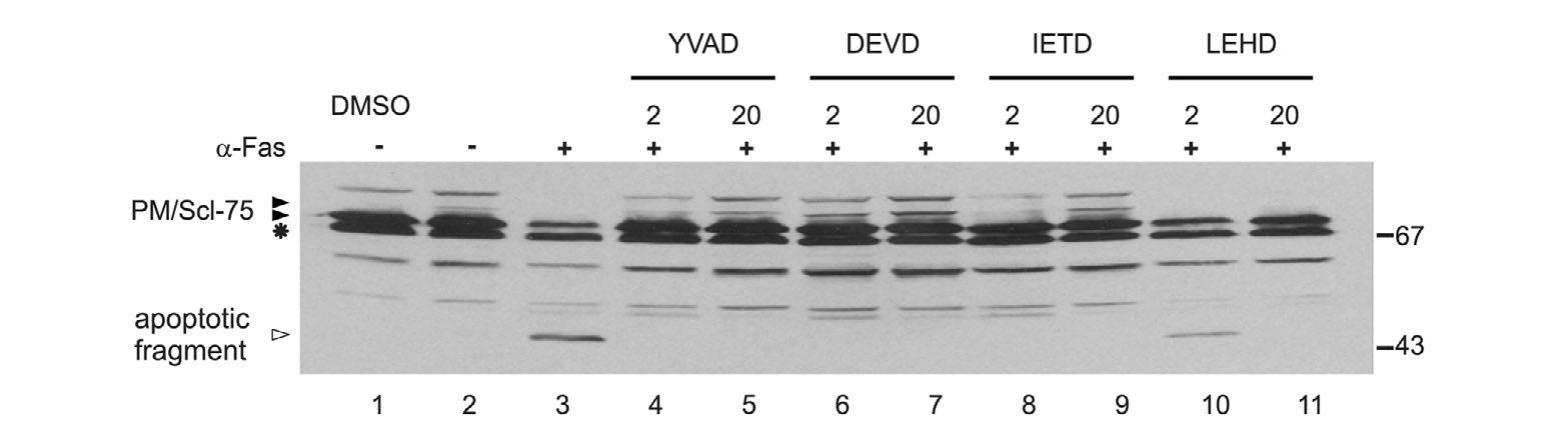
Inhibition of PM/Scl-75 cleavage by caspase inhibitors. Jurkat cells were cultured for 1 hour at 37°C in the presence of four cell-permeable tetrapeptide caspase inhibitors: 2 μM (lanes 4, 6, 8 and 10) or 20 μM (lanes 5, 7, 9 and 11) inhibitors of caspase-1 (YVAD), caspase-3 (DEVD), caspase-8 (IETD) and caspase-9 (LEHD). Subsequently, cells were cultured for 8 hours in the presence of anti-Fas monoclonal antibody (α-Fas). Control extract from mock-treated Jurkat cells was loaded in lane 1. In lanes 2 and 3 extracts from control and apoptotic (8 hours anti-Fas mAb) Jurkat cells, respectively, were analyzed. Total protein extracts were analyzed by 10% SDS-PAGE and Western blotting with the anti-PM/Scl-75 rabbit serum. The positions of molecular mass markers are indicated in kDa at the right and the positions of the relevant polypeptides at the left. Filled arrowheads indicate full-length proteins, open arrowheads indicate cleavage products and the asterisk indicates cross-reactivity of the anti-PM/Scl-75 serum.

Because the activation of caspases is a common feature of apoptotic cells and because activated caspases are capable of cleaving a large number of (autoantigenic) proteins, we investigated whether caspase activation was required for cleavage of PM/Scl-75. Jurkat cells were cultured in the presence of various inhibitors of caspases for 1 hour before the induction of apoptosis by anti-Fas. Four different tetrapeptide caspase inhibitors were used, namely Ac-YVAD-CMK for group I caspases (caspase-1-like), Z-DEVD-FMK for group II caspases (caspase-3-like), and Z-IETD-FMK (caspase-8) and Z-LEHD-FMK (caspase-9) for group III caspases. Rabbit anti-PM/Scl-75 antiserum was used to analyze the cleavage of PM/Scl-75 in these cells. The cleavage of PM/Scl-75 was completely inhibited in the presence of 2 or 20 μM Ac-YVAD-CMK, Z-DEVD-FMK or Z-IETD-FMK (Figure 4, lanes 4 to 9). In the presence of Z-LEHD-FMK, PM/Scl-75 cleavage was partly inhibited at 2 μM and completely inhibited in the presence of a 10-fold higher concentration (Figure 4, lanes 10 and 11). As a control for the inhibitory activity of the tetrapeptide inhibitors, the cell extracts were also analyzed for cleavage of U1-70K and Sm-F. The inhibition of cleavage of both caspase substrates seemed to be very similar to that observed for PM/Scl-75 (data not shown). These results suggest that caspases, or other proteases activated downstream of the caspase cascade, are involved in the apoptotic cleavage of PM/Scl-75.

### Cleavage of PM/Scl-75 by different caspases

**Figure 5:**
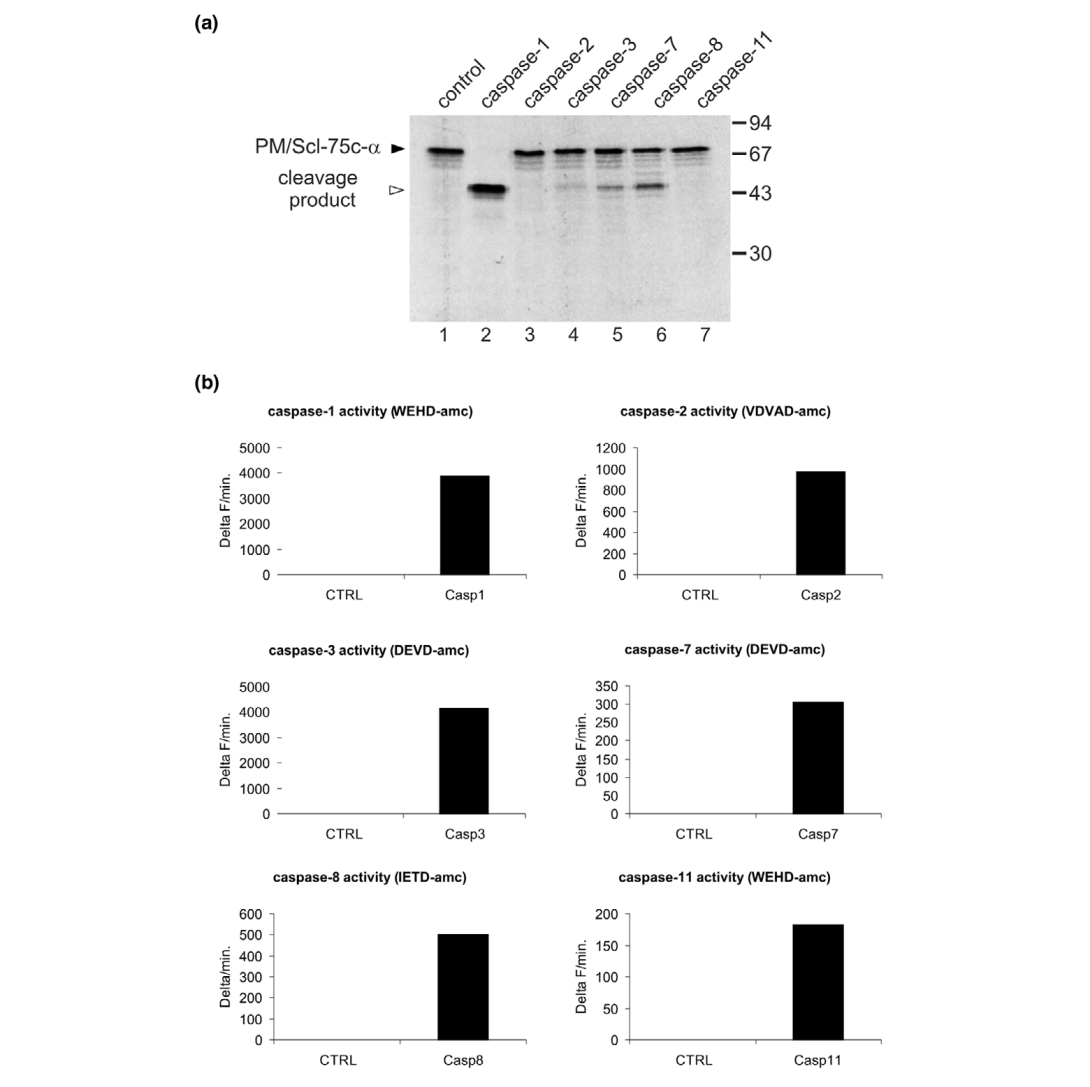
Cleavage of PM/Scl-75 by recombinant caspases. **(a) **^35^S-labeled PM/Scl-75 translated *in vitro *was incubated with 200 nM purified murine recombinant caspase-1, caspase-2, caspase-3, caspase-7, caspase-8 or caspase-11 for 1.5 hours at 37°C. The resulting reaction products were analyzed by 10% SDS-PAGE, followed by autoradiography. In the first lane the mock-incubated protein was loaded. The filled arrowhead marks the full-length proteins and the open arrowhead the cleavage products. Note that in this experiment the pCI-neo-VSV-PM/Scl-75c-α cDNA was used for *in vitro *transcription and translation of PM/Scl-75. The positions of molecular mass markers are indicated in kDa at the right and the positions of the relevant polypeptides at the left. **(b) **As a control for caspase activity the recombinant caspases were incubated with 50 μM fluorogenic substrate in 200 μl of CFS buffer. The release of fluorescent 7-amino-4-methylcoumarin was monitored for 1 hour at 37°C at 2-minute intervals in a fluorimeter. Data are expressed as the increase in fluorescence (Delta F) as a function of time.

To determine whether PM/Scl-75 is a direct substrate for caspases, PM/Scl-75 translated *in vitro *was incubated with recombinant murine caspase-1, caspase-2, caspase-3, caspase-7, caspase-8 and caspase-11. Four of these caspases seemed to be able to cleave PM/Scl-75, resulting in cleavage products similar in size to the fragment generated *in vivo *(Figure 5a). However, the efficiencies by which this protein is cleaved by the recombinant caspases showed marked differences. Caspase-1 very efficiently cleaved PM/Scl-75, whereas decreasing efficiencies were observed for caspase-8, caspase-7 and caspase-3. The recombinant caspase-2 and caspase-11 did not result in detectable levels of PM/Scl-75 cleavage (Figure 5a, lanes 3 and 7). As a control for caspase activity, all recombinant caspases were incubated with a specific fluorogenic substrate (Figure 5b). Because caspase-8 has a key role in apoptosis mediated by a death domain receptor, we tested whether caspase-8 is essential for the cleavage of PM/Scl-75 in apoptotic cells: caspase-8-deficient Jurkat cells (ATCC CRL-2571) were used to analyze the apoptotic processing of PM/Scl-75. These cells become apoptotic when treated with staurosporin in combination with cycloheximide. Despite the absence of caspase-8, on induction of apoptosis the same cleavage pattern was observed as in control cells containing caspase-8 (data not shown). This suggests that, in agreement with the proteolysis results *in vitro*, other caspases contribute to the cleavage of PM/Scl-75 in apoptotic cells.

### Identification of the caspase cleavage site in PM/Scl-75

The association of the 45 kDa PM/Scl-75 fragment with the exosome (see above) suggested that this fragment represents the N-terminal part of the protein, because this region contains the RNase PH domain, which is involved in the association with the exosome complex [14]. Immunoprecipitations performed with caspase-1-cleaved *in vitro*-translated PM/Scl-75 carrying a VSV-G tag at either the N or C terminus did indeed show that the 45 kDa fragment could be precipitated only when the VSV tag was attached to the N terminus of the protein (data not shown). These data indicate that the major caspase cleavage site in PM/Scl-75 is located in the C-terminal domain of the protein.

**Figure 6:**
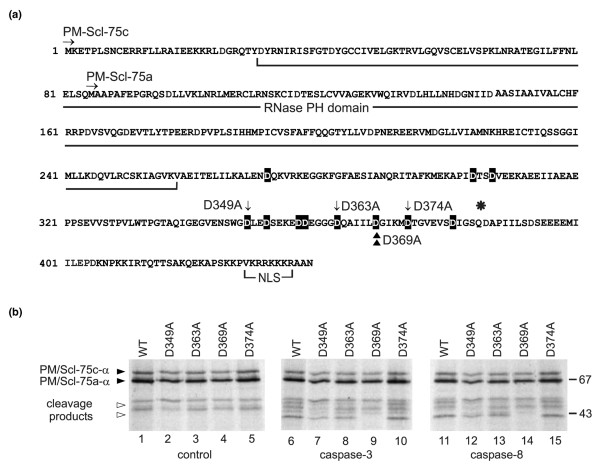
Mapping of the caspase cleavage site in PM/Scl-75. **(a) **Amino acid sequence of PM/Scl-75 (α variant). The RNase pleckstrin homology (PH) domain, the nuclear localization signal, the N termini of both published sequences, PM/Scl-75a [13] and PM/Scl-75c [14], and the position (asterisk) where the β splice variant contains a 17-amino-acid insertion are indicated. Note that the numbering of the amino acid positions corresponds to that of PM/Scl-75c-α. All aspartic acid residues that were replaced by alanines are highlighted in black. The mutants that were used in the experiment shown in (b) are marked by arrows (D349A, D363A and D374A) and the identified cleavage site by arrowheads (D369A). **(b) **Cleavage of PM/Scl-75 mutants by recombinant caspases. *In vitro *translated ^35^S-labeled PM/Scl-75c-α and mutants of the protein in which the aspartic acid residues at positions 347 (D347A), 363 (D363A), 368 (D369A) or 374 (D374A) were changed into alanine residues were incubated with purified murine recombinant caspase-3 or caspase-8 for 1.5 hours at 37°C. The resulting reaction products were analyzed by SDS-PAGE followed by autoradiography. In lanes 1 to 5 the proteins incubated in parallel in the absence of caspases were loaded (control). The positions of molecular mass markers are indicated in kDa at the right, and the positions of relevant polypeptides are shown at the left. Filled arrowheads indicate full-length PM/Scl-75c-α and open arrowheads indicate the PM/Scl-75 cleavage product. Note that in this experiment the pCR4-TOPO-PM/Scl-75c-α cDNA [14] was used for the transcription and translation of PM/Scl-75 *in vitro*. As a result of translation initiation at an internal start codon in the reticulocyte lysate, a relatively high level of a second polypeptide was produced, which was shown to represent a polypeptide initiated at Met85, thus corresponding to PM/Scl-75a-α. WT, wild type.

To determine the exact position of the cleavage site in PM/Scl-75, substitution mutants were generated in which the aspartic acid residues marked in Figure 6a were replaced by alanine residues. Aspartic acid residues were chosen because each of the caspases requires this amino acid at the cleavage site, and thus replacement by alanine is expected to interfere with cleavage. After incubation with the recombinant caspase-3 or caspase-8, the specific cleavage product was no longer observed with the PM/Scl-75 protein containing the substitution at position 369 (D369A) (Figure 6b, lanes 9 and 14), whereas the other mutations did not influence the cleavage of PM/Scl-75 by these caspases. In addition, when this mutant was incubated with caspase-1 the cleavage of PM/Scl-75 was abolished (data not shown). Taken together, these data suggest that the IILD^369^↓G sequence represents the caspase cleavage site in the PM/Scl-75 protein during apoptosis.

## Discussion

Although many different autoantibodies have been identified in a variety of autoimmune diseases, the mechanism that leads to the production of most of these autoreactive antibodies is still unknown. During the past decade a substantial number of autoantigens have been shown to be modified during apoptosis and/or necrosis. This has led to the hypothesis that intracellular, modified autoantigens are exposed to the immune system because of massive cell death and/or inefficient removal of dying cells, which could elicit a primary immune response targeting the modification on the autoantigen. In a secondary response, other parts of the autoantigenic molecule or its interacting partners might also be targeted by the immune system as a result of epitope spreading [1]. Patients with the PM/Scl overlap syndrome often develop antibodies against several components of the human PM/Scl or exosome complex, especially against PM/Scl-100, PM/Scl-75 and hRrp4p [19]. Here we show for the first time that one of the exosome subunits, PM/Scl-75, is specifically modified during apoptosis. The generation of a PM/Scl-75 fragment in apoptotic lysates of caspase-8-deficient Jurkat cells, the results from the caspase inhibitor studies in anti-Fas-treated cells, and the cleavage of PM/Scl-75 by different caspases *in vitro *resulting in a similar cleavage pattern all suggest that several caspases are implicated in the proteolytic cleavage of PM/Scl-75. However, we cannot exclude the possibility that cleavage of PM/Scl-75 is not performed directly by caspases and is instead an indirect effect of the activation of other proteases during apoptosis. The cleavage of PM/Scl-75 occurs in the C-terminal part of the protein at the unconventional caspase cleavage site IILD^369^↓G. However, cleavage of other autoantigens such as DNA topoisomerase I and Sm-F have also been reported to be cleaved by caspases at unconventional sites [16,20,21].

Consistent with the observation that the C-terminal part of the protein is cleaved during apoptosis, leaving the RNase PH domain intact, is our finding that a subset of cleaved PM/Scl-75 molecules remains associated with the core of the exosome complex (Figure 3). A very similar situation has been described for the Sm-F protein, which is cleaved apoptotically while remaining associated with the heptameric ring of the Sm complex [16]. On the basis of interactions between exosome components and structural similarity with the bacterial protein polynucleotide phosphorylase, a model was generated for the structure of the human exosome. In this model the six proteins containing an RNase PH domain form the core of the exosome, which adopts a hexameric ring structure [22]. This model is strongly supported by the crystal structures of the archaeal, yeast and human exosome [23-26]. Interestingly, the crystal structure of the human exosome shows that the C-terminal extension of PM/Scl-75 interacts with hRrp46 and wraps around both hRrp46 and OIP2 (hRrp43) on the outer surface of the ring, demonstrating that the identified cleavage site of PM/Scl-75, which is located in the middle of this extension, is accessible to caspases. The observation that PM/Scl-75 is cleaved during apoptosis might also have implications for the activity and function of the exosome complex.

Recently, it has been reported that the activity of the human exosome is restricted to the hRrp41p–PM/Scl-75 heterodimer. As a consequence, cleavage of PM/Scl-75 may influence the catalytic activity of the complex [26]. Moreover, it has been shown that PM/Scl-75 contains a nuclear localization signal, which has a role in the nucleolar targeting of PM/Scl-75 [14]. The cleavage of PM/Scl-75 in apoptotic cells removes the nuclear localization signal from the protein, which may change the subcellular distribution of the protein and the associated complex, as has been reported for the La autoantigen [27]. It is therefore tempting to speculate that cleavage of PM/Scl-75 leads to an increased exosome concentration in the cytoplasm and/or in the nucleoplasm, which may be required for an enhanced degradation rate of a variety of RNA molecules in these compartments during apoptosis. Simultaneously, cleavage of PM/Scl-75 would result in the release of the exosome from the nucleolus, leading to a loss of exosome function in this cellular compartment.

As described above, recent studies have led to the hypothesis that cell-death-induced modifications can generate neo-epitopes that trigger an autoimmune response. If the immune system is exposed to elevated and persistent levels of apoptotically modified PM/Scl-75, this could contribute to breaking the immunological tolerance to the exosome complex, leading to the production of autoantibodies against components of this complex. It would therefore be interesting to investigate whether the B-cell repertoires of PM/Scl patients in the early phase of the disease contain antibodies that are specifically reactive with the apoptotic PM/Scl-75 protein fragment. Such autoantibodies that preferentially recognize apoptotically modified isoforms of the autoantigen have been shown to exist for the U1-70K protein [28,29].

## Conclusion

This study shows that the autoantigenic exosome component PM/Scl-75 is specifically cleaved during apoptosis. A 45 kDa fragment of PM/Scl-75 is generated in apoptotic Jurkat cells. This fragment, which corresponds to the N-terminal part of PM/Scl-75, is associated at least in part with the exosome complex. Cleavage assays *in vitro *with different recombinant caspases suggest that several caspases might be responsible for the proteolytic cleavage of PM/Scl-75, although caspase-1 seems to be the most effective. The caspase cleavage site was mapped in the C-terminal part of the protein at Asp369 (IILD^369^↓G).

## Abbreviations

VSV-G = vesicular stomatitis virus G epitope.

## Competing interests

The authors declare that they have no competing interests.

## Authors' contributions

GS, RR, XS and GJMP participated in the design of the study. GS, RR, KCRM, WVE and LVW performed the experiment and data analysis. GJMP, XS, PV and WJvV supervised the project. All authors read and approved the final manuscript.
